# Analysis of the association between paternity and reoperation for urethral obstruction in adult hypospadias patients who underwent two-stage repair in childhood

**DOI:** 10.1186/s12894-019-0512-2

**Published:** 2019-10-04

**Authors:** Akihiro Kanematsu, Shiro Tanaka, Takahiko Hashimoto, Michio Nojima, Shingo Yamamoto

**Affiliations:** 1 Department of Urology, Hyogo College of Medicine, Mukogawacho 1-1, Nishinomiya, Hyogo 663-8501 Japan; 2 Department of Clinical Biostatistics, Graduate School of Medicine Kyoto University, Yoshida Konoecho, Sakyo, Kyoto, 606-8501 Japan

**Keywords:** Epidemiology, Hypospadias, Paternity, Surgery, Urethral obstruction

## Abstract

**Background:**

The association between surgical outcome of hypospadias repair and long-term male reproductive function has not been documented. The purpose of this study was to clarify association between paternity in adult hypospadias patients and reoperation for urethral obstruction after two-stage repair during childhood.

**Methods:**

Ninety hypospadias patients who underwent the same kind of two-stage repair in our institute by a single surgeon, were initially treated at < 18 years old, and who were ≥ 18 years old during the survey were included in the study. Present physical, social, and life status were evaluated by a mailed self-entry questionnaire, and clinical background and surgical outcome data were evaluated by medical records. National survey data of the general population were used as external control. The paternity rate of the patient groups was evaluated by Kaplan-Meier curve analysis and log-rank tests.

**Results:**

Twenty-six patients (28.9%) underwent 43 reoperations after completion of the initial repair. Twelve patients were reoperated for obstructive complication (Study group) and were compared with 14 patients who were reoperated only for non-obstructive causes and 64 patients who were not reoperated as Study control group (*N* = 78). The Study group patients showed sexual intercourse rate and marriage rate not statistically different in comparison with the Study control, although marriage rate at 32.5 years old were lower than the general population (*p* = 0.048, z-test). None of the Study group achieved paternity, which showed a significant difference to the Study control (*p* = 0.032, log-rank test). The difference was also statistically significant in the analysis among the 31 married patients (*p* = 0.012, log-rank test). Patients reoperated for obstructive complication documented worsened Quality of Life score in the International Prostate Symptom Score (2.3 ± 2.0 vs. 1.4 ± 1.2, *p* = 0.031, t-test) and ejaculation problems (66.7% vs. 17.4%, *p* = 0.003, chi-square test).

**Conclusions:**

History of reoperation for obstructive complication was associated with lower paternity rate in patients with hypospadias, presumably for multifactorial causes associated with marriage age and ejaculation problems. The present results may implicate importance of uncomplicated urethroplasty during childhood for achieving paternity, although it should be further tested in the future for larger groups of hypospadias patients.

**Electronic supplementary material:**

The online version of this article (10.1186/s12894-019-0512-2) contains supplementary material, which is available to authorized users.

## Background

Hypospadias is a defect in penile development. The natural history of mild hypospadias is unclear but could have a mostly benign course without treatment [1]. However, patients with more severe hypospadias undergo repair during infancy, in the hope that with corrected penis, the patient will subsequently have a normal reproductive function as a male. However, long-term consequences in patients with repaired hypospadias have been unclear.

Population based studies have reported decreased paternity in hypospadias patients, but precise disease and surgical background were not implicated in those studies [2, 3]. Several groups have recently conducted long-term follow-up studies, [4–9] and a Swedish group reported that sexual function and fertility of adult patients with hypospadias are equivalent to those of their age-matched controls but inferior in proximal type patients [7]. In our previous report, we introduced a survival analysis method for analyzing the outcome of patients with hypospadias operated at our institute. We also reported that patients with hypospadias in general had equivalent intercourse and marriage rates compared with the general population [10]. Our study also revealed that while sexual intercourse, marriage, and paternity occurred in serial order, overlap was scant in the factors associated with these three events. The intercourse rate was lower in patients with proximal hypospadias, and the marriage rate was lower in those without stable jobs. These were expected findings, but were statistically proven for the first time using multivariate survival analysis.

An unexpected finding in that study was the lower paternity rate in those who underwent reoperation after the initial repair. We did not analyze this finding further, because that study had included patients who had undergone diverse surgical procedures including two-stage repair, one-stage repair, and reoperative procedures after multiple repairs. Therefore, in this study, we analyzed the paternity data of our hypospadias patient cohort, exclusively limited to those who underwent the same initial two-stage repair operation in relation to surgical complications requiring reoperation. We hypothesized that reoperation for urethral obstruction after initial hypospadias repair could be related with decreased paternity, and aimed to clarify the association between them.

## Methods

### Subjects

Patients with hypospadias operated between 1973 and 1998 by a single surgeon at our institution were candidates for this study. During this period, no other surgeon performed hypospadias repairs at our institution. Two-stage repair was the preferred procedure. In the first stage, the chordee tissue surrounding the penile shaft was completely removed and glandular urethra was created by inverted preputial flap. In the second stage, the skin between glandular urethra and original meatus was tubularized using a method like the Johansson technique. This procedure was the mainstay for most hypospadias repairs for 25 years at our institution, with the exception of very mild glandular cases, for which, other techniques such as meatal advancement and glanuloplasty-incorporated procedures were performed [11].

### Data collection

This study was approved by the Ethics Committee of the Hyogo college of Medicine (approval number 1147). We contacted the parents of the candidate patients by postal mail and asked them to forward a self-entry questionnaire and an informed consent form to the patients. In addition to the International Prostate Symptom Score (IPSS) [12] and the International Index of Erectile Function-5 (IIEF-5), [13] the survey included questions by Moriya et al., regarding the patients’ educational and social history, sexual and marital experience, paternity, present complaints with respect to penile shape, hesitation before and after the first intercourse, and difficulties during intercourse and ejaculation, as described in the Additional file 2 [14]. History of additional surgery for hypospadias outside our institute was also obtained. Patients who had undergone a two-stage repair in our institute at < 18 years of age, were ≥ 18 years during the current survey, and had returned evaluable responses were included in the study. All study participants provided written informed consent. Those with documented sex development disorders, two with mixed gonadal dysgenesis and one with XX male, were excluded. As was done in our previous study, glandular and penile type hypospadias were compared with proximal type. Data of birth weight, gestational age at birth, hypospadias type, presence of undescended testes, and additional surgical procedures performed after repair completion were extracted from the patients’ clinical records. National survey data of general population were used as external control to assess sexual intercourse and marriage rates, which were respectively defined as the percentage of the male population in the country that have had sexual intercourse and been married at least once [15].

### Main outcome measures

**Fig. 1:**
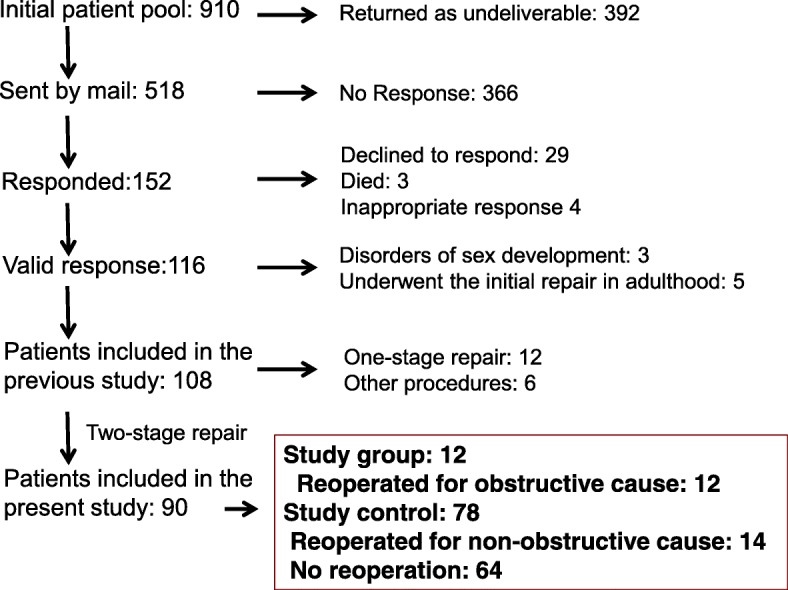
A diagram showing selection of the study patients from the initial candidate pool

Patients were initially divided into three groups, those with no reoperation and those reoperated for either non-obstructive or obstructive complications. Patients with a history of at least one reoperative procedure for obstructive causes were considered as reoperated for obstruction. Based on the findings described in the Additional file 1: Figure S1, patients having no reoperation and those reoperated only for non-obstructive cause were used as Study control group for the Study group of patients reoperated for obstructive causes (Additional file 1: Figure S1 and Fig. 1).

### Statistical analyses

Kaplan–Meier curves were used for estimating rates of masturbation, heterosexual intercourse, marriage, and paternity. The intercourse and marriage rates were compared with the age-specific rates reported in a national survey of the general population, using z-tests with Kaplan–Meier estimates and their standard errors. Analyses for paternity were performed using the age at marriage of the patients with offspring, as the precise birth time of their children had not been asked in the questionnaire. The comparison between the subdivided groups was performed using a log-rank test on Kaplan–Meier curves, and comparison with General population was performed by z-tests, with *p* < 0.05 considered to be significant. Chi-square test and t-test were used to assess the differences between the groups. All statistical analyses were performed using JMP 9 software (SAS Institute Inc., Cary, NC, USA).

## Results

### Study population

Our initial pool consisted of 910 potential participants, with 392 (43.0%) questionnaires returned undelivered. Of the remaining 518 patients, 152 patients (28.4%) returned the completed questionnaires, as reported in our previous study [10]. Of these, 90 patients with a history of the same two-stage repair by the single surgeon [16] were included in this study (Fig. 1).

### Development ladder of male sexual life

**Fig. 2:**
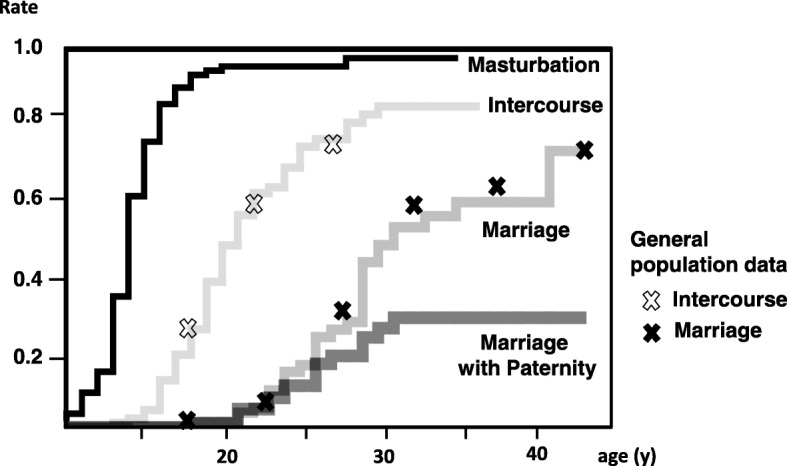
Developmental ladder of male sexual activity in the study patients. Developmental ladder of male sexual activity in the study patients is demonstrated as Kaplan–Meier curves. Most of the patients experienced masturbation by the end of puberty. Intercourse and marriage rates of the general population as reported in the national survey are plotted by X. The intercourse and marriage rates were not statistically different to the control data (z-test) [10]. Paternity is plotted as date of marriage of the patients with offspring

Development of male sexual activities is plotted in Fig. 2. Patients with hypospadias are known to experience sexual desire and begin masturbation during puberty similar to other males, [9, 14] which was a finding confirmed by our study cohort. The experience rates of both sexual intercourse and marriage also increased along with age. Consistent with the findings of our previous study, the rates were equivalent to the general population data derived from the national survey [10, 15]. Paternity rates also increased with participant age but did not equal marriage rates.

### Classification and timing of reoperation

**Fig. 3:**
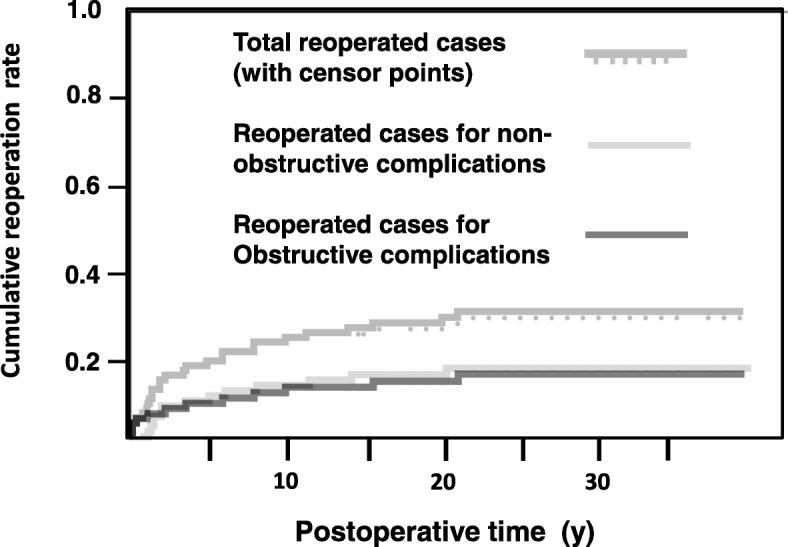
Cumulative incidence of reoperation. Cumulative incidence of total reoperation (*n* = 26), reoperation for obstructive complications (*n* = 12), and reoperation for non-obstructive complications (*n* = 14) is plotted as Kaplan–Meier curves. Patients who had at least one procedure for obstructive causes were classified as reoperated for obstructive complications. For the patients undergoing multiple reoperations, event points were plotted as the date of first reoperation after the initial repair. Reoperative procedures decreased along with time but were performed for nearly 20 years postoperatively. The occurrence of reoperation was nearly identical between the patients operated for obstructive and non-obstructive complications

We classified reoperated participants based on whether the underlying urethral complication was obstructive or non-obstructive. In total, 26 patients (28.9%) underwent 43 reoperative procedures, of which 18, 24, and 1 were for obstructive, non-obstructive, and unknown causes, respectively (Table 1). The cumulative experience of reoperation after the initial repair completion is plotted in Fig. 3. The overall reoperation rate was < 20% at 2 years, as previously reported,(16) but continued to increase over the years. The timing of the first reoperation was similar between the patients operated for either obstructive (*n* = 12) or non-obstructive (*n* = 14) complications. The age of the last reoperation was 13.8 ± 8.2 y for those with obsructive complications, and 9.6 ± 5.5 y for those with non-obstructive complications (*p* = 0.063).

**Table 1:** Complication and time until reoperative procedures

Complication	Procedure	Case	Procedure	Post-operative time to procedure (mo)				
				Median	Min	Max	Mean	SD
Obstructive		12	18	41.5	3	120	86.2	41.5
Urethral stricture	Internal urethrotomy	7	12	44	3	120	44	42.9
	Urethral dilation	1	1	2	NA	NA	NA	NA
Meatal stenosis	Meatal cutback	2	2	15.5	3	28	NA	NA
Urethral dilatation	Reduction urethroplasty	3	3	96	17	252	NA	NA
Not obstructive		19^a^	24	30	6	190	76.7	75
Meatal regression	Meatoplasty	12	14	98	11	190	102.2	87.5
Fistula	Fistula closure	6	7	26	6	93	44.4	37.8
	Cutback to fistula	1	1	28	NA	NA	NA	NA
Recurrent bending	Chordectomy	1	1	18	NA	NA	NA	NA
	Repeat Urethroplasty		1	30	NA	NA	NA	NA
Unknown	Unknown	1	1	240	NA	NA	NA	NA
Total		26	43	41.5	3	332	82.2	41.5

^a^ Including 5 cases who also had obstructive complications. One case had 1 fistula closure and 1 meatoplasty

*SD* Standard deviation

*NA* Not applicable

### Reoperation for obstructive complication is associated with a lower paternity rate

**Fig. 4:**
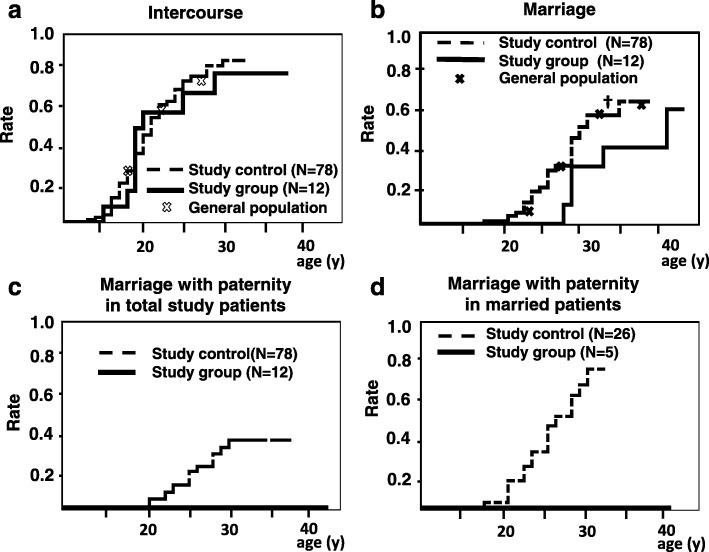
The cumulative rate of sexual intercourse, marriage, and paternity. **a** and **b** The cumulative rate of sexual intercourse and marriage is shown, stratified as having been reoperated for obstructive complication (Study group) or others (Study controls), together with national survey data (General population, plotted by X). **a** No statistical difference was noted in the intercourse rate (*p* = 0.76) between the Study group and Study controls. There was no significant difference with the General population either. **b** The marriage rate of the Study group at 32.5 years old was significantly lower than that of the General population (†: *p* = 0.048, z-test). At 27.5 years old, the marriage rate of the General population and Study group was radically different, 36.7% vs. 0%, although *p*-values of the z-test could not be calculated because there was no event in the Study group before these time points. *P*-value for the difference in the marriage rate between the Study group and Study controls was not statistically significant in the log-rank test (*p* = 0.13). **c** and **d** No paternity was observed in patients reoperated for obstruction. Paternity rates were significantly lower in patients reoperated for obstruction in analysis of total patients (**c**, *p* = 0.032 by log-rank test) and in analysis of married patients (**d**, *p* = 0.012 by log-rank test)

We, performed survival analysis of the patients. Those reoperated for obstructive causes were defined as Study group (n = 12). The non-reoperated cases (*n* = 64) along with the patients reoperated only for non-obstructive causes (*n* = 14) were grouped together as the Study control group (*n* = 78). The sexual intercourse rate was statistically equivalent between the Study group and the Study controls (Fig. 4a). However, in comparison with that in the General population, the age at marriage was equivalent in the Study controls, but significantly higher in the Study group at 32.5 years old (†: *p* = 0.048, z-test). Notably, no patients in the Study group had offspring. The difference was statistically significant both in the analyses among the total patients (Fig. 4c, *p* = 0.032 by log-rank test) and among the 31 married patients (Fig. 4d, *p* = 0.012 by log-rank test). The two cohorts of Study control group, patients without reoperation and patients reoperated only for non-obstructing complications showed similar curve in either analysis (Additional file 1: Figure S1A and B).

### Clinical and social data of the patients stratified by presence and type of reoperation

Clinical and social data of the patients are listed in Table 2. While the gestational age at birth was lower in the Study group than in the Study controls, the birth weight was not significantly different. Interestingly, distribution of hypospadias type was similar among those who had reoperation and those who did not. No difference was noted in the rate of concomitant undescended testes either. The mean age at the time of survey and the follow-up time were significantly longer in the Study group than in the Study controls. Job status, intercourse rates, and marriage rates were equivalent between both groups. Although the Study group patients tended to marry older, hesitation before and after the first intercourse experience was not significantly higher than the Control.

**Table 2:** Clinical and social data of the patients

		Total	Study Control			Study Group (Reoperated for obstruction)	*P*-value
			No	Reoperated	Control		
			reoperation	No obstruction	Sum		
Number		90	64	14	78	12	
Gestational age at birth (weeks)		38.0 ± 3.1	38.3 ± 2.8	36.9 ± 3.1	38.2 ± 2.86	35.5 ± 5.1^a^	0.015
Birth weight (g)		2764 ± 783	2707 ± 812	2921 ± 733	2750.5 ± 760.5	2789 ± 863	0.88
Hypospadias type	Grandular and Penile	44 (0.49)	31 (0.48)	7 (0.50)	38(0.49)	6 (0.50)	1
	Proximal^b^	34 (0.38)	24 (0.38)	6 (0.43)	30(0.38)	4 (0.33)	
	Unknown	12 (0.13)	9 (0.14)	1 (0.07)	10(0.13)	2 (0.17)	
Undescended testis		9 (0.10)	7 (0.11)	1 (0.07)	8(0.10)	1 (0.08)	1
Operated age (y)		4.3 ± 2.9	4.6 ± 3.3	3.3 ± 1.6	4.3 ± 3.1	4.0 ± 1.7	0.74
Age at the time of survey (y)		28.7 ± 6.9	27.7 ± 8.1	27.5 ± 7..5	27.7 ± 6.4	34.8 ± 7.4^a^	0.0061
Follow up time (y)		24.3 ± 6.6	23.2 ± 5.9	24.2 ± 7.2	23.3 ± 6.1	30.8 ± 6.7^a^	0.0001
Stable job	n (rate)	57 (0.63)	42 (0.66)	7 (0.50)	49 (0.63)	8 (0.67)	1
Intercourse	n (rate)	77 (0.74)	43 (0.67)	8 (0.57)	51 (0.65)	9 (0.75)	0.74
Marriage	n (rate)	31 (0.34)	21 (0.33)	5 (0.36)	26 (0.33)	5 (0.42)	0.75

^a^ Statistically significant compared with the Study control by t-test (*p* < 0.05)

^b^ Proximal: Penoscrotal, Scrotal and Perineal Type

### Persistent difficulties in micturition and ejaculation in patients reoperated for urethral obstruction

Present symptoms of the patients were evaluated through a mailed questionnaire (Table 3). The IPSS-QOL score revealed impaired micturition status in the Study group compared with the Study controls. However, the difference between the two groups for the total IPSS as well as for each IPSS domain and subdomains (subdivided into either obstructive or non-obstructive symptoms) was not statistically significant. This may indicate that the impaired IPSS-QOL score may be related to micturition issues not expressed by specific IPSS questions.

**Table 3:** Questionnaire data of the patients

	Study Control			Study group (Reoperated for obstruction)	*P*-value
	No	Reoperated	Control		
	reoperation	No obstruction	Sum		
Number	64	14	78	12	
IPSS total	3.5 ± 3.8	3.9 ± 4.5	3.6 ± 3.9	4.9 ± 5.6	0.31
IPSS-QOL	1.4 ± 1.2	1.4 ± 1.2	1.4 ± 1.2	2.3 ± 2.0	0.037^a^
IIEF-5 Q1	3.2 ± 0.9	3.6 ± 1.1	3.3 ± 0.9	3.3 ± 1.0	1
Married patients	21 (0.33)	5 (0.36)	26 (0.33)	5 (0.41)	0.75
Patients with paternity	10 (0.16)	3 (0.21)	13 (0.16)	0 (0.0)	0.2
IIEF-5 total (for married patients only)	20.6 ± 4.2	21.2 ± 2.3	20.7 ± 3.9	20.1 ± 2.9	0.75
Problems (Yes/Response (Rate))					
Penile shapes	33/64 (0.52)	9/14 (0.64)	42/78 (0.54)	5/11 (0.45)	1
Penile bending	7/64 (10.9)	2/14 (0.14)	9/78 (11.5)	1/11 (0.90)	0.78
Intercourse	13/49 (0.27)	0/8 (0.00)	13/57 (0.23)	4/9 (0.44)	0.22
Difficult intercourse for penile bending	1/45	0/8	1/53	0/9	1
Ejaculation	11/63 (0.17)	2/14 (0.14)	13/77 (0.17)	6/9 (0.67)	0.0042^b^
Weak or incomplete ejaculation	10/63 (0.16)	2/14 (0.14)	12/77 (0.16)	6/9 (0.67)	0.0017^b^
Hesitation for intercourse (Yes/Response (Rate))					
Before first intercourse	19/55 (0.35)	1/12 (0.08)	20/78 (0.30)	4/10 (0.40)	0.34
After first intercourse	10/44 (0.27)	0/8 (0.00)	10/52 (0.19)	3/8 (0.38)	0.48

^a^ Statistically significant compared between Study group and control by t-test (*p* < 0.05)

^b^ Statistically significant compared between Study group and control by Fischer’ exact test (*p* < 0.05)

The patients reported problems associated with sexuality by responding to IIEF-5 and questionnaires by Moriya et al.,[14] which included questions asking whether patients had problems with penile shape, sexual intercourse (i.e., if they had experienced any), and ejaculation. Patients were also asked whether they experienced any hesitation before and after their first intercourse experience. No statistically significant difference was noted in IIEF-5 Q1 nor in questions about penile shape and intercourse. The problem of bending was documented by 9/78 (11.5%) in control, and 1/11 (9.0%) in study (*P* = 0.78, not significant). It was problematic for sexual intercourse in only one patient in Control group. The main issue reported was that the patients had to squeeze the semen out during ejaculation, but none documented anejaculation. The only significant finding was that a higher proportion of the Study group had problems with ejaculation than that of the Study controls, specifically for weak or incomplete ejaculation (66.7% vs. 15.6%, *p* = 0.0017). Therefore, ejaculation difficulties were the only subjective problem significantly associated with a history of urethral reoperation for obstructive causes. These results may suggest that urethral obstruction have caused ejaculatory problems in the Study group patients.

## Discussion

This study reports that reoperation for obstructive complications after the initial pediatric urethroplasty was associated with a decreased paternity rate in adult hypospadias patients who had undergone a two-stage procedure at our institution. This type of study, linking a childhood surgical outcome with the adult potential for paternity, is scarce in literature.

This study was conducted as a sub-analysis of our previous report, which unexpectedly revealed that urethral reoperation was related to decreased paternity. Out of 108 patients in our earlier study, we selected out 90 patients who underwent a two-stage repair by a single surgeon from mild to severe type of hypospadias. Thus, we could analyze the surgical results without being confounded by differences in the surgical procedure. The two-stage repair was the mainstay for most of the hypospadias patients at the time. Among the 518 mailed patients with the exclusion of 80 cases in which operative procedure was not identified, 81.7% (358/438) underwent the same two-stage repair, which is equivalent to 83.3% (90/108) of the present and previous studies. This may indicate that our responders represent the total patient group treated during that period without significant selection bias with regard to the operation procedure.

We analyzed the type of reoperations the patients underwent and found that reoperations have been performed for more than 20 years after the initial repair. Such accumulation of reoperations in longer studies has also been reported by other authors [17]. This finding could explain why the final reoperation rate was as high as 28.9% in the surgeon’s series who documented less than 10% reoperation rate in up to 5 years of follow-up [16]. The timing of urethrocutaneous fistula repair after the initial repair was relatively shorter than that of the other types of reoperation. The total fistula rate, 7.8% (7/90), was similar to the short-term result of the same surgeon’s series, which reported a fistula rate of around 7.9–8.9% for all degrees of hypospadias [16]. Reoperations for other two major complications, urethral obstruction and meatal regression, typically occurred after a longer interval. Interestingly, in our patients, the complication rates between the proximal cases and the non-proximal cases were similar, presumably due to the uniformity of the procedure.

The most important finding in this study was that in a sharp contrast to that in the Study controls, paternity was absent in the Study group patients who were reoperated for obstructive complications of the urethra, despite the longer follow-up period and the higher age of the participants at the time of study. This is a novel finding, as paternity data in hypospadias patients have not previously been associated with a surgical outcome during childhood in the existing literature. While one relevant article from Sweden reported lower paternity in patients with proximal hypospadias, the surgical complications were not implicated as a cause, and the patients in that study had undergone diverse surgical procedures by multiple surgeons [7].

The cause of lower paternity in our Study group patients may be multifactorial. The z-test revealed that the marriage rate of the Study group at 32.5 years old was significantly lower than that of the General population, implying that these patients married later in life. We cannot infer whether reoperation for urethral obstruction delayed their marriage, either for psychological or physical reasons. Mureau et al. reported that, the later the patients underwent surgery, the greater were their inhibitions in seeking sexual contact and the later they made the first sexual contacts [18]. However, between the patients reoperated for obstructive or non-obstructive cause, the timing of the first and last reoperation was not statistically significant, nor was the age of first intercourse.

As another point, a later marriage may have reduced the child-bearing period, with presumably more aged partner that those who married earlier. There were 5 married patients in the study group, all of whom did not achieve paternity. Since we did not ask them whether they had attempted to have children, we cannot conclude whether they were infertile or simply elected to remain childless. It is plausible that urethral obstruction had an adverse effect on sperm emission, since the Study group documented difficulties with ejaculation at a rate significantly higher than the Study controls. Since 4 out of the 5 married Study group patients underwent only palliative surgery such as 3 internal urethrotomy and 1 urethral dilatation, they might still have had urethral obstruction persisting into adulthood. However, we lack the seminalysis, uroflow, and concrete penile shape data, which is a major limitation of our study, as we relied mainly on questionnaires to collect data. One interesting point is that, after exclusion of the patients reoperated for obstructive causes, the final paternity rate of the married patients in the Study controls reached nearly 80% on the Kaplan–Meier curve (Fig. 4d), which is equivalent to that of the general population at 83.3% [15]. We may thus infer that if there had been no obstructive pathology that required reoperation, the final paternity rate of hypospadias patients might have been closer to that of the general population.

Because this study is a sub-analysis of our previous study, it has the same kind of limitation as have been already documented in the earlier report. One drawback, inevitable in this type of study, was the limited response rate to the questionnaires, although our response rate at 28.4% was comparable to that in other similar studies [6, 14]. Limited response can cause selection bias, but we would like to note that there was no significant difference in background between the responders and non-responders with respect to hypospadias severity as reported in the previous study [15] and the type of initial repair procedure as described in this report. Another limitation is that the majority of the information regarding the present condition of the participants was obtained through a survey, and there is a lack of objective data, such as seminalysis or uroflowmetry. Since our data derive from limited number of patients, with 12 Study group patients without offsprings, of which only 5 were married, our findings should be further tested by future study by different groups and by ourselves. To overcome this drawback in such future studies, we may have to maintain a prospective follow-up protocol up to adulthood when we discharge our patients from outpatient visit today. Lastly, the data were obtained from patients who underwent a hypospadias repair surgery different from more recent procedures, and the patients were generally older at the time of initial repair than today. Over the last two decades, surgery for hypospadias correction has shifted to different types of one-stage repair, with the urethral plate as the preferred material compared to preputial skin used earlier [19, 20] and patients tend to be treated at a younger age. Although current repair like tubularized incised plate method may have better results than the procedures performed in this article, twenty to thirty year follow up should be needed to obtain final outcome, as described in this report.

## Conclusions

The findings in this report may suggest that reoperation for urethral obstruction was associated with lower paternity, but because of the limited number of Study patients, it should be further tested by the future studies in larger groups of hypospadias patients. Nonetheless, the present results may implicate importance of uncomplicated urethroplasty during childhood for achieving paternity and may be a warning message from the past for current and future hypospadias surgeons.

## Additional files

Additional file 1: **Figure S1.** The cumulative rate of paternity in reoperated patients with or without obstruction compared with patients without reoperation. A. and B.: Fig. 4c and d are repotted with Study control data subdivided into two cohorts, patients without reoperation and patients reoperated only for non-obstructing complications. Note that the two cohorts of the Study control show similar curve in either analysis. (PPTX 79 kb)

Additional file 2: Excerpts from Hypospadias-specific questionnaire (from Moriya et al. [14]). (DOCX 20 kb)

## Data Availability

The datasets for this article are available in the data base repository of Hyogo College of Medicine or from the corresponding author upon request.

## Abbreviations

IIEF-5: International Index of Erectile Function
IPSS: International Prostate Symptom Score
IPSS-QOL: IPSS Quality of Life Score
