# Supplementary material for: Analysis of the association between paternity and reoperation for urethral obstruction in adult hypospadias patients who underwent two-stage repair in childhood
Source: BMC Urol. 2019 Oct 4;19:88. doi: 10.1186/s12894-019-0512-2 (PMC6778371; doi:10.1186/s12894-019-0512-2)
Supplement: Supplementary file 1 — Figure S1. The cumulative rate of paternity in reoperated patients with or without obstruction compared with patients without reoperation. A. and B.: Fig. 4c and d are repotted with Study control data subdivided into two cohorts, patients without reoperation and patients reoperated only for non-obstructing complications. Note that the two cohorts of the Study control show similar curve in either analysis. (PPTX 79 kb) [file 12894_2019_512_MOESM1_ESM.pptx]

## Slide 1
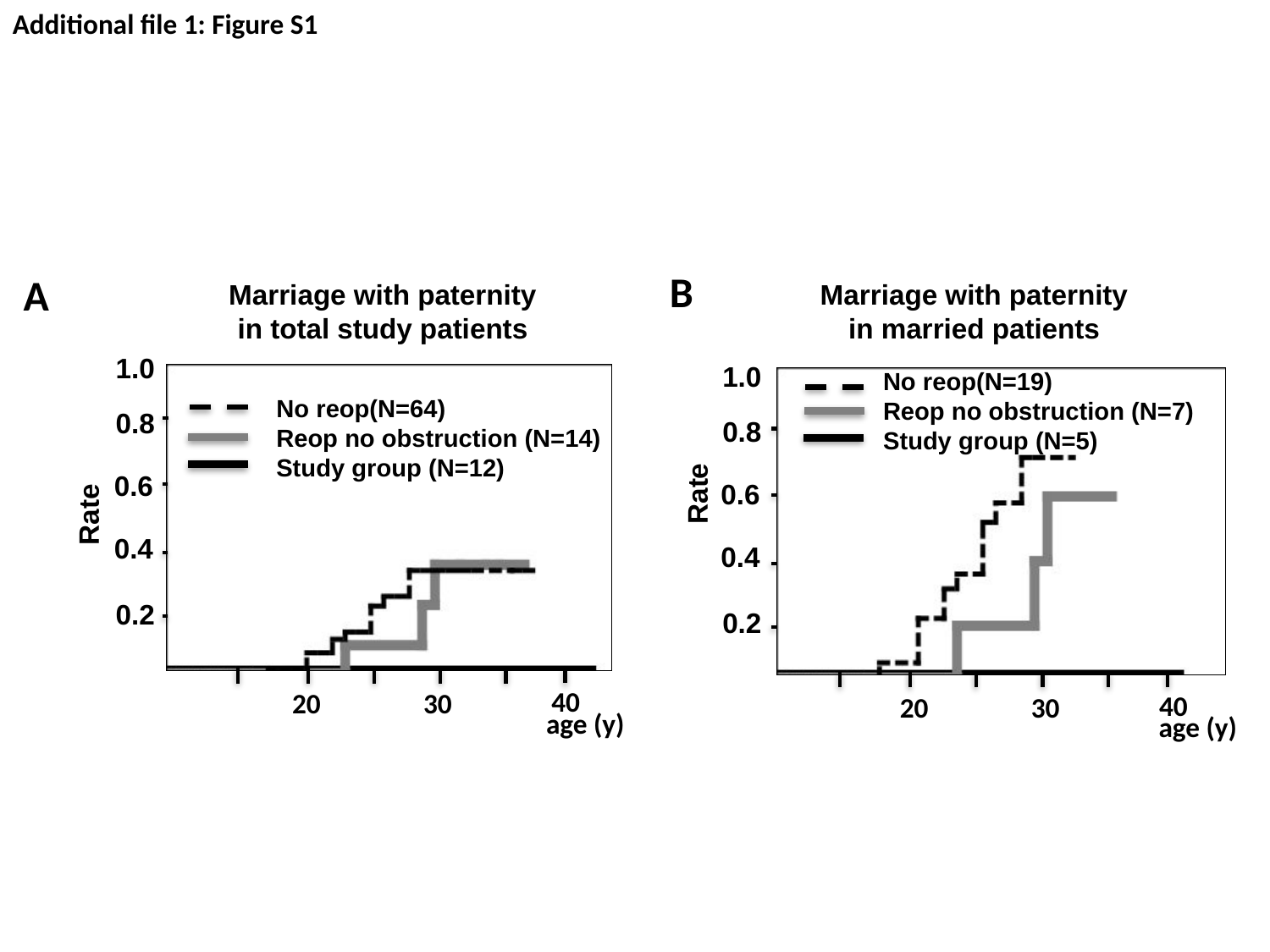

Additional file 1: Figure S1
B
A
Marriage with paternity
in total study patients
Marriage with paternity
 in married patients
1.0
0.8
0.6
0.4
0.2
1.0
0.8
0.6
0.4
0.2
No reop(N=19)
Reop no obstruction (N=7)
Study group (N=5)
No reop(N=64)
Reop no obstruction (N=14)
Study group (N=12)
Rate
Rate
40
20
30
age (y)
40
20
30
age (y)
