# Supplementary material for: Analysis of the association between paternity and reoperation for urethral obstruction in adult hypospadias patients who underwent two-stage repair in childhood
Source: BMC Urol. 2019 Oct 4;19:88. doi: 10.1186/s12894-019-0512-2 (PMC6778371; doi:10.1186/s12894-019-0512-2)
Supplement: Supplementary file 2 — Excerpts from Hypospadias-specific questionnaire (from Moriya et al. [14]). (DOCX 20 kb) [file 12894_2019_512_MOESM2_ESM.docx]

**Additional file 2**

Excerpts from Hypospadias-specific questionnaire (from Moriya et al. 2006)^14^

Q. 1. What is your final school experience?

1. Junior high school, graduated
2. High school, not graduated
3. High school, graduated
4. Trade school, not graduated
5. Trade school, graduated
6. College/university, not graduated
7. College/university, graduated
8. Other (detailed description )

Q. 2. What is your present job?

1. Student
2. Agriculture
3. Deskwork
4. Physical work
5. No stable job
6. Specialized job
7. Other (detailed description )

Q. 3. Have you ever been married with a woman or lived as a couple in a way similar to marriage?

1. Yes 2. No

Q. 3-1. If yes, at what age did it begin? years old

Q. 6. Do you have a biological child?

1. Yes 2. No

Q. 7. What is your present height and weight?

Height cm Weight kg

Q. 17 Are you unsatisfied with your penis?

1. Yes 2. No

Q17-1 If yes, what is the reason for your dissatisfaction?

1. Phimosis
2. Lack of prepuce
3. Too small size
4. Too large size
5. Position of meatus
6. Penis itself
7. Shape of the glans
8. Presence of scar
9. Color of the Penis
10. Bending of the penis
11. Hair of the penis
12. Others (Please specify )

Q. 26. Since the time you underwent surgery at our institution, have you ever undergone a reoperation performed by the urology or pediatric surgery department of another institution?

1. Yes 2. No

Q. 29 Do you have any problem with ejaculation?

1. Yes 2. No 3. I have never experienced ejaculation

Q29-1 If you answered ‘Yes’ to Q.29, what is your problem with ejaculation?

1. No semen comes out.
2. I have to squeeze my penis to bring out semen.
3. Painful ejaculation
4. Semen sprays around
5. Others (please specify)

Q30 Before you experienced sexual intercourse, have you felt hesitation for sexual activity because of your penile shape?

1. Yes 2. No

Q. 31. Have you ever had sexual intercourse (=sex) with a female?

1. Yes 2. No

Q. 31-1. If yes, when did you have your first sexual intercourse experience?

years old

Q31-4 After you experienced sexual intercourse, have you felt hesitation for sexual activity because of your penile shape?

1. Never
2. Hesitation has disappeared
3. Hesitation persists
4. Hesitation became worse

Q31-5 Do you have any problem in having sexual intercourse (=sex)

1. Yes 2. No

Q31-6 If you answered ‘Yes’ to Q31-5, what is the problem for intercourse.

1. Penis is not rigid enough
2. Erection does not persist
3. Pain during intercourse (=sex)
4. Others (Please specify )
